# Supplementary material for: Deletion of wfs1 Impairs Oligodendrocyte Precursor Cells Dorsal Distribution and Myelination Through the wfs1-hmgcs1 Axis in Zebrafish
Source: Biology (Basel). 2026 Aug 16;15(16):1402. doi: 10.3390/biology15161402 (PMC13510151; doi:10.3390/biology15161402)
Supplement: Supplementary file 1 [file biology-15-01402-s001.zip › File S1-Transcriptome sequencing data and differentially expressed genes (DEGs) in wfs1-- zebrafish at 5 dpf/2.KEGG/wfs1KOvsWT/UP/wfs1KOvsWT.up_KEGGdot.pdf]

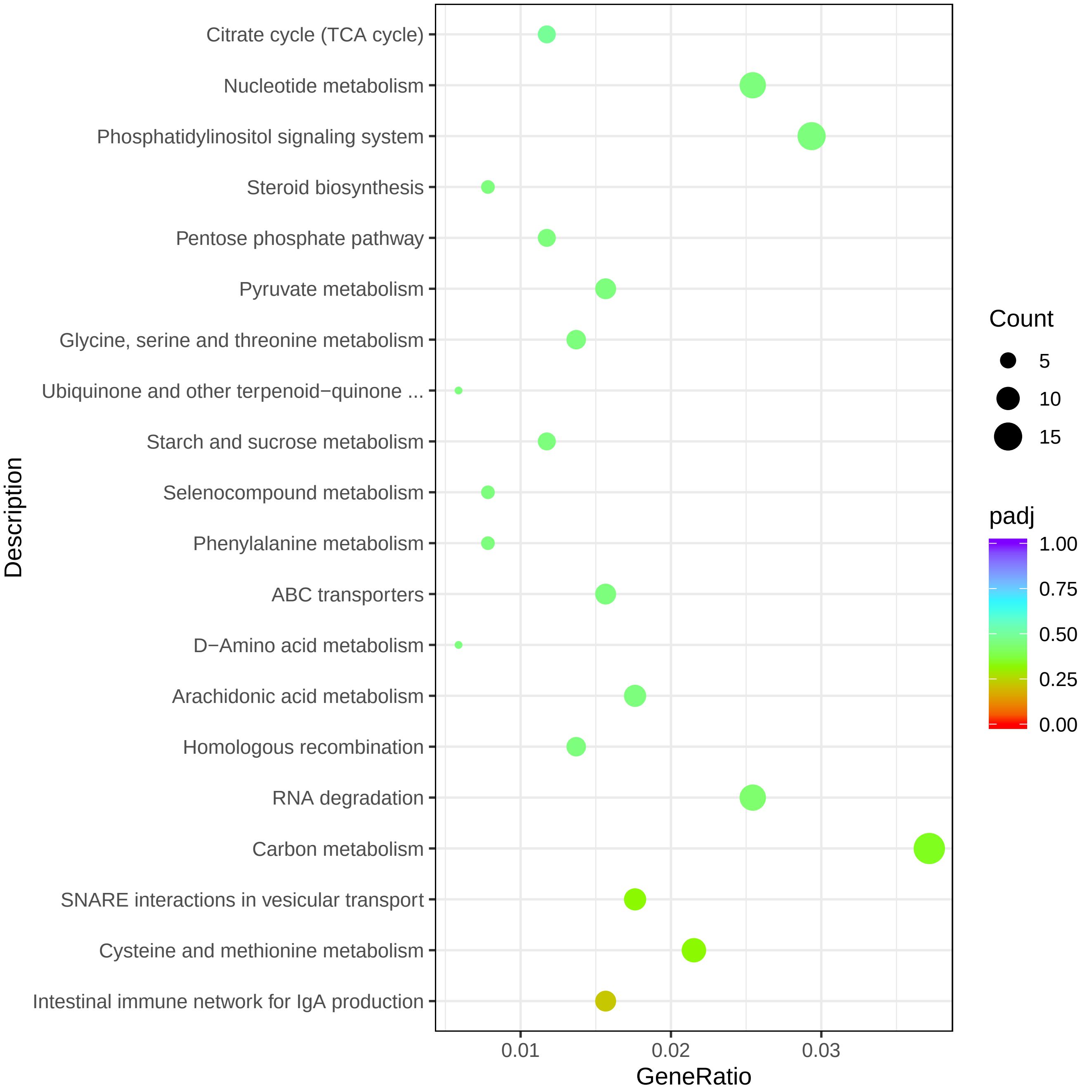
Citrate cycle (TCA cycle)
Nucleotide metabolism
Phosphatidylinositol signaling system
Steroid biosynthesis
Pentose phosphate pathway
Pyruvate metabolism
Glycine, serine and threonine metabolism
Ubiquinone and other terpenoid−quinone ...
Starch and sucrose metabolism
Selenocompound metabolism
Phenylalanine metabolism
ABC transporters
D−Amino acid metabolism
Arachidonic acid metabolism
Homologous recombination
RNA degradation
Carbon metabolism
SNARE interactions in vesicular transport
Cysteine and methionine metabolism
Intestinal immune network for IgA production
Description
0.01
0.02
0.03
GeneRatio
Count
5
10
15
padj
1.00
0.75
0.50
0.25
0.00
